# The Effect of Virtual Fencing Stimuli on Stress Responses and Behavior in Sheep

**DOI:** 10.3390/ani9010030

**Published:** 2019-01-21

**Authors:** Tellisa Kearton, Danila Marini, Frances Cowley, Susan Belson, Caroline Lee

**Affiliations:** 1 School of Environmental and Rural Science, University of New England, Armidale, NSW 2350, Australia; danila.marini@csiro.au (D.M.); fcowley@une.edu.au (F.C.); caroline.lee@csiro.au (C.L.); 2 Commonwealth Scientific and Industrial Research Organization, Agriculture and Food, Locked Bag 1, Armidale, NSW 2350, Australia; sue.belson@csiro.au

**Keywords:** Animal welfare, avoidance learning, behavior, body temperature, cortisol, ear postures, electric shock, livestock, stress, vigilance

## Abstract

**Simple Summary:**

Virtual fencing is a new technology that uses audio signals and electrical stimuli to spatially control animals without the need for fixed fencing. It involves avoidance learning whereby the animals learn to respond to an audio cue (conditioning stimulus) to avoid receiving an aversive electrical stimulus (unconditioned stimulus). The audio cue is used to warn the animal that it is approaching the boundary and should be benign and not perceived as aversive to the animal. While a positive punishment stimulus is necessary for learning, it should not be so aversive to the animal that it impinges on its welfare. This study aimed to determine how the stimuli used in virtual fencing are perceived by the animal when they are first encountered. The audio and electrical stimuli were compared to other commonly encountered stimuli in normal sheep production systems, including a barking dog and a restraint procedure. The physiological and behavioral responses of sheep indicated that sheep were no more adversely impacted by virtual fencing stimuli than they were by other commonly encountered stimuli. The least to most aversive treatments were: Control < Beep < Barking Dog < Electrical stimulus < Restraint.

**Abstract:**

To understand the animal welfare impact of virtual fencing stimuli (audio cue ‘beep’ and electrical stimulus) on naïve sheep, it is necessary to assess stress responses during the animal’s first encounters with these stimuli. Eighty Merino ewes were exposed to one of the following treatments (*n* = 16 animals per treatment): Control (no stimuli), beep, dog bark, manual restraint, and electrical stimulus. Collars were used to apply the audio and electrical stimuli. The restraint treatment showed an elevated cortisol response compared with the control (*p* < 0.05), but there were no differences between the other treatments and the control. There were no differences between treatments in vaginal temperature (*p* > 0.05). For behaviors, the sheep receiving the bark and beep treatments were more vigilant compared to the control (*p* < 0.05), there were more aversive responses observed in the electrical stimulus treatment compared to the control. Together, the responses showed that the beep stimuli were largely benign, the bark stimuli was minimally aversive, the electrical stimuli was acutely aversive, and the restraint was moderately aversive. These data suggest that, for sheep, their first exposure to the virtual fencing stimuli should be perceived as less aversive than a commonly used restraint procedure.

## 1. Introduction

Virtual fencing for the containment of sheep has the potential to significantly alter many common practices, with potential to improve grazing and pasture management in livestock production systems. Traditional fencing systems such as mesh and electric are used extensively in many countries around the world, particularly in Australia and the United States, where animals are grazed over large distances. This fencing is expensive and time consuming to maintain, and vulnerable to damage from adverse weather events. In these cases, virtual fencing provides producers with not only a more cost effective and low maintenance solution for the containment of stock, but also an effective way to move stock from one area to another, simply by moving the fence-line. The successful implementation of virtual fencing relies on the principles of classical conditioning and associative learning to allow the animal to learn that an audio stimulus is associated with a subsequent electrical stimulus (ES). The audio stimulus is applied as the animal approaches the fence and the electrical stimulus is only applied if the animal continues to move forward into the fence boundary, as described by Lee et al. [1], Lee [2], and Campbell et al. [3]. A commercial virtual fencing product has been developed for cattle (eShepherd^TM^) [4]. There is currently research underway to develop a similar product in sheep [5].

Virtual fencing utilizes two stimuli, one audio and one electrical stimulus, which are novel experiences for untrained animals, and therefore have no contextual association upon first exposure. The animal welfare impacts of this technology in sheep are unknown, and therefore, a comparison with other commonly encountered husbandry stressors is proposed. In the context of welfare assessment, the application of new technologies may be assessed via a new welfare assessment framework developed by Lee et al. [6], in which predictability and controllability (P/C) may influence positive or negative welfare outcomes in animals. At the beginning of virtual fence training, the sheep’s encounters with the aversive electrical stimuli are low in both predictability and controllability, and there is potential for poor welfare if this state is prolonged [7]. As the animal learns to respond to the presumed benign audio signal, they are able to learn to predict and control their encounters with the aversive electrical stimulus, and this will ultimately impact on the valence of their welfare state [8].

Utilizing the framework described in Lee, Colditz, and Campbell [6], it is possible to assess the welfare and stress responses associated with the initial learning phase, when the stimuli are both unpredictable and uncontrollable, to determine the relative aversiveness of the audio cue and the electrical stimulus in comparison with other commonly experienced husbandry practices. Measures of stress associated with electrical stimuli used in virtual fencing have been investigated in cattle [9], and it was found that the stress response to the electrical stimulus was similar to the response to being restrained in a crush. Similar work has not yet been conducted in sheep and no work has investigated the initial responses to the audio cue. 

While traditional electric fences have been used for some time in production systems, there appears to be little research conducted on the stress responses of animals upon their first encounters with an electrical stimulus. Sheep experience a variety of auditory and physical stimuli both aversive and benign throughout their lives in normal production systems. Many routine management practices, such as shearing, are known to be stressful for sheep [10]. Sheep have evolved to flee from predators and therefore they are flighty and may be sensitive to unnatural cues. Sheep have a highly developed auditory system, similar to that of dogs [11], and as a result, may be sensitive to aversive audio stimuli. Dogs are widely used in sheep production and research has shown that the presence of a barking dog is aversive to sheep [12,13,14]. It is likely that this aversion is partially related to the sound of the dog barking and partially to the physical presence of the dog, and previous experiences and contexts are likely to shape the strength of the aversive response.

The aims of this study were to investigate initial stress responses to the audio and electrical stimuli used in virtual fencing in comparison with other stimuli commonly encountered by the animals in commercial sheep production systems. By using naïve animals with no prior experience of virtual fencing stimuli, we sought to emulate the experiences of sheep upon their initial encounters with virtual fencing cues, to give an indication of how sheep perceived the stimuli at the beginning of the learning process when the stimuli were both unpredictable and uncontrollable. These impacts were assessed using commonly used measures of stress: Plasma cortisol [10,15,16], body temperature [17,18,19], and behavioral responses [13,20,21]. We predicted the stress response to the audio cue would be minimal and not different to the control treatment of no stimuli and that the response to the electrical stimulus would be similar to the barking dog and less aversive than one-min of restraint. Overall, we predicted that the least to most aversive treatments may be ranked as: Control, beep, bark, electrical stimulus, and restraint.

## 2. Materials and Methods 

The experiment was undertaken at CSIRO’s McMaster Laboratory, Armidale, New South Wales (NSW), Australia. The protocol and conduct of the experiment was approved by The CSIRO Chiswick Animal Ethics Committee under the NSW Animal Research Act, 1985 (approval ARA 17/26).

### 2.1. Animals

Ninety Merino non-pregnant ewes (mean body weight 38.5 kg ± 0.41 kg) aged between 3 and 5 years were introduced to the animal house and fed standard rations of 200 g blended chaff and 700 g complete pelleted ration (Ridley Agriproducts, Australia; 9.04 MJ/kg dry matter) per animal per day, and provided with water ad-libitum These sheep had been exposed to standard industry practices such as restraint for shearing and herding with dogs. The sheep were housed in large pens (4 × 4 m) in groups in the animal house for initial habituation, and were moved to individual pens (2 × 1 m) in an adjacent animal house facility for additional training and during the testing periods.

The sheep were randomly allocated to one of five treatments which were tested in four cohorts *n* = 20 each that were habituated to handling and movement to and from the testing area, and tested sequentially. Five spare animals were included in habituation in the first and second cohorts and three in the third and fourth cohorts, two spare animals were returned to the paddock as it was decided that they would not be needed at that stage. 

### 2.2. Habituation 

To commence habituation, the first two cohorts were moved into individual pens, under a covered shed which was open on the north face. The sheep were housed in close proximity to other individuals which allowed visual and social interaction. All sheep were fitted with dummy collars for the duration of the habituation period (14 days) to acclimate them to the weight of the electric collars and to ensure that all sheep were subject to the same habituation conditions. Sheep undergoing treatments requiring active collars had their collars changed over immediately, following baseline blood samples being taken on test days. All sheep were handled twice daily and restrained manually in a standing position for 20 s to simulate blood sample collection and to minimize the interference of stress associated with this procedure during the experiment. To habituate the sheep to the test arena, they were moved in treatment groups of four into the arena where they stayed for 1 minute and then moved back into their pens twice daily for two weeks, the same sheep were moved together each time to simulate test day procedure. Habituation and testing movements were conducted in groups of four so as to minimize the stress response to social isolation. Following habituation and the completion of the testing, the first two cohorts of sheep were returned to their paddocks and the third and fourth cohorts were moved into the individual pens to commence habituation and testing as described for cohorts one and two, following which they were also returned to their paddocks. All the sheep had their necks shorn to facilitate blood sampling, this was performed the day prior to testing so that any stress responses associated with this procedure would have fully resolved before testing commenced. 

### 2.3. Experimental Design and Treatments

From the 90 sheep in all cohorts, 80 were randomly selected for inclusion in the experiment. These 80 sheep were randomly allocated to one of five treatments: 1) control―no stimuli, 2) beep, 3) bark, 4) restraint, and 5) electrical stimulus (ES), *n* = 4 per treatment per cohort. The beep and bark were audio treatments and the restraint and electrical stimulus were physical treatments. Testing of treatments occurred on the second day following completion of the habituation period, with cohorts one and two tested on consecutive days, and cohorts three and four tested on consecutive days following their habituation period. Four animals from each treatment were tested together on each day, totalling 16 animals per treatment over the course of the experiment, and treatment order randomized for each cohort. Each animal was exposed to one treatment only. For application of the treatments, the sheep were moved in treatment groups of 4 through a raceway into the test area, which consisted of a room approx. 2 × 2 m that had been covered in insulating material so as to reduce the travel of the sounds from the box to the untested animals outside. Once each treatment group was moved to the test area, they remained there for one minute prior to treatment commencing, and were released from the test area approximately 30 s following the completion of the treatment. Treatment details were as follows:

1. Control animals entered the testing area for 1 min and were not subjected to stimuli.
2. Beep treatment animals were fitted with remotely controlled Garmin collars prior to entry to the testing area and the beep tone (45–55 dB, 2.7 kHz) was manually applied to each of the four collared animals simultaneously three times for two seconds with a two-second interval in between, two experimenters each operated two collar control devices. The duration was based upon the virtual fencing protocol developed by Marini, Meuleman, Belson, Rodenburg, Llewellyn, and Lee [5].
3. Bark treatment involved playing the sound of a dog barking (58–68 dB, 6.1 kHz) through a speaker placed centrally in the testing area, with 3 s played for 2 s each with an interval of 2 s in between.
4. Restraint treatment involved handlers entering the test area and each catching one sheep by hand (one handler per sheep). The sheep was then placed in an inverted restraint position (ventral belly exposed) for a period of 60 s, such as sheep would experience undergoing normal shearing practices.
5. Electrical stimuli (ES) treatment animals were fitted with Garmin dog control collars (Garmin TT15, Garmin Ltd., Kansas, KS, USA) placed snugly around the neck below the jaw, ensuring that the probes were in close contact with the skin. To ensure good contact the animals in this treatment group had their necks shorn more extensively the day prior to testing. Three electrical stimuli were applied to each of the four collared animals simultaneously for approximately 500–600 milliseconds each, with intervals of two seconds in between each application, with two operators as per beep treatment. The electrical stimulus was set to level 4 (320 V, 20 us, 16 pulses per/sec) out of a possible 18. The level chosen has been identified in previous studies as being sufficiently aversive to achieve the desired conditioned response, but not so aversive as to cause negative behavioural responses such as jumping and running around [5]. The level chosen is much lower than traditional electric fencing, in which industry standards recommend 4000–5000V for sheep [22], and lower than that used on sheep in previous research by Brunberg et al. [23] at 4000V and Martin et al. [24] at 6500V. The intensity of the electrical stimulus used was also lower than that used in a similar experiment conducted by Lee, Fisher, Reed, and Henshall [9] on cattle at 600V.

Shortly following the treatment (<1 min) animals were returned to their individual pens. The time interval between leaving and returning to the home pen was approximately 5 min. 

One animal was removed from the trial due to illness prior to the testing day and was treated accordingly and returned to the paddock. It was replaced with a spare animal that had undergone the same acclimation and handling procedures.

### 2.4. Body Temperature and Blood Sampling

Body temperature is a common measure in the detection of stress in sheep [17,18,19] and vaginal temperature measures were chosen for this purpose. The day prior to testing days the sheep were fitted with a Thermochron iButton^©^ (Model number DS1922L-F5, accuracy 0.5 °C, resolution 0.063 °C, weight 3.3 g; Maxim International, San Jose, CA, USA) temperature logging device fitted to a intravaginal controlled drug release device previously leached of drug actives (CIDR^®^, Zoetis, Parsippany, NJ, USA) using polyolefin heat-shrink tubing, as described by Lea et al. [25]. Data were extracted using the program eTemperature version 8.32 (OnSolution, Castle Hill, Australia). Loggers were set to record body temperature in increments of 10 s beginning at 7 am on testing days and were fitted to the animal the day prior in order to remove any effects of restraint during fitting. The loggers were removed at the end of the testing day. 

Blood sampling for plasma cortisol was performed, as plasma cortisol is a useful measure of acute stress in sheep [10,15,16]. On testing days each sheep was restrained and baseline blood samples were taken via jugular venepuncture into 10 mL EDTA coated Vacutainer tubes within 1 min and immediately prior to movement to the treatment area. Additional blood samples were taken at 10 min, 20 min, 30 min, 60 min, 120 min, and 240 min following the treatment. Blood samples were centrifuged at 3000 rpm for 10 min at 4 °C, and plasma was retained and stored at −18 °C for analysis. Samples were analyzed for plasma cortisol concentration using a commercial radioimmunoassay (Plasma Cortisol RIA, MP Biomedicals, California, CA, USA). This method has been previously validated in our laboratory for use in sheep [26]. The intra-assay and inter-assay coefficients of variance (CV) for quality controls containing 24.9 nmol/L, 51.6 nmol/L, and 104.9 nmol/L of cortisol were 2.3%, 2.1% and 3.0% and 23.0%, 14.4%, and 16.7%, respectively.

### 2.5. Behavioral Analysis

A number of commonly used behavioral measures were used in the detection of stress responses in sheep. These included vigilance [21], ear posture [20], and locomotor activity [27], as well as some unusual and novel behaviors observed. The behaviors measured are described and categorized in Table 1. Video footage was recorded by two handheld video cameras (Sony Handycam HDR-XR550, Sony Electronics Inc., San Diego, CA, USA) which were set up inside the treatment area to capture behavioral data during and after the application of treatments. Sheep behavior was analyzed using Observer XT^©^ 12.0 software (Noldus Ltd., Wageningen, The Netherlands) to collate specific behaviors using video collected from the treatment area. The behaviors are described in Table 1. 

Observations were made for two measurement periods: The treatment period lasting 10 s encompassing the time the treatments were applied, and a twenty second period following the treatment, referred to as “post-treatment”. The control observation period was also split into these two measurement periods to allow an equivalent comparison, however there was no difference in the treatment across these two periods. Continuous behaviors were analyzed as a proportion of time spent, expressed as a percentage; while point behaviors were analyzed as count events. Behaviors were not able to be recorded for the restraint treatment as once animals were caught minimal behaviors were shown, and when released the presence of handlers may have impacted on behaviors and so animals were allowed to move out of the treatment area immediately following the restraint. Point behaviors were pooled into characterizations (as described in Table 1) for statistical analysis.

### 2.6. Statistical Analysis

Statistical analysis was performed in R [28] using the packages nlme [29], pscl [30], MASS [31], rcompanion [32] and userfriendlyscience [33]. Vaginal mean and peak temperature data met assumptions of normality using visualisation of Q–Q plots and the Shapiro-Wilk test of normality (Shapiro-Wilk). Analyses of mean and peak body temperature (for each animal from time 0–60) were performed using a linear mixed effects model with four levels of cohort included in the model as an interaction term and individual sheep as a random effect. Time series temperature data did not meet assumptions of normality and could not be improved by transformation and was subsequently analyzed with the non-parametric Kruskal-Wallis rank sum test and the Jonckheere-Terpstra test. This was used for both whole data sets and across individual time points. Post-hoc multiple comparison tests were also performed to account for any family-wise error. Temperature measures for these tests were characterized as 5 min averages for 60 min from treatment time for analysis, with baseline measurements at 10 min prior to treatment. Additionally, area under the curve data and individual time points were analyzed (time 0 min, 10 min, 20 min, 30 min, 40 min, 50 min, and 60 min from treatment), as well as change from baseline (time 0 min, 10 min, 20 min, 30 min, 40 min, 50, and 60 minutes from treatment). These data did not meet assumptions of normality and could not be improved with transformation and were subsequently analyzed using the non-parametric Kruskal-Willis test. Raw data are provided in the Supplementary Materials.

Plasma cortisol data were log_e_ transformed and subsequently met assumptions of normality (Shapiro–Wilk). Analysis was performed by one way repeated measures analysis of variance (ANOVA) using a linear mixed effects model with individual sheep as a random effect, and time 0 as a covariate. Mean, peak and change in cortisol were analyzed using a linear mixed model with cohort included in the model as an interaction term and individual sheep as a random effect. There was no significant interaction between treatment and cohort and so the interaction term was removed and the day was included in the model as a fixed effect. A linear mixed model with ANOVA was also used for time series plasma cortisol analysis. There was no significant interaction between time and treatment, or between cohort and treatment, so the interaction terms were removed and time and cohort were included in the model as fixed effects. Cortisol concentration curves had all returned to baseline by 60 min from treatment time and so this period was used for analysis. Area under the curve analysis was also performed for plasma cortisol 0–60 min using a linear mixed model with cohort included in the model as an interaction term and individual sheep as a random effect. There was no significant interaction between treatment and cohort and so the interaction term was removed and the cohort was included in the model as a fixed effect.

The duration of display of vigilance behavior was analyzed as a percentage of the observation time spent, and were tested for normality using visual assessment of Q–Q plots and the Shapiro-Wilk test of normality. These data did not meet assumptions of normality or equal variance and could not be improved through transformation. Analysis of vigilance was consequently performed using the Kruskal-Wallace non-parametric one-way analysis of variance (ANOVA) along with a multiple comparison test. The duration of time in each ear posture was analyzed as a percentage of observation time. These data also did not pass tests for normality or equal variance and therefore a Kruskal-Wallis rank sum test, Jonckheere-Terpstra test and multiple comparison tests were used, looking at differences between proportions of time spent in each ear posture across the treatments. The frequency of ear posture change was analyzed by a linear mixed model with the cohort included in the model as a fixed effect and individual sheep as a random effect. Normality assumptions were met using visualization of Q–Q plots and the Shapiro-Wilk test of normality. The number of steps taken during the observation periods was analyzed using Poisson regression. Other count behaviors were converted to binary measures and analyzed using Fisher’s exact test with post-hoc pairwise tests of independence for nominal data used to investigate treatment differences. 

## 3. Results

### 3.1. Physiological Measures

#### 3.1.1. Plasma Cortisol

**Figure 1:**
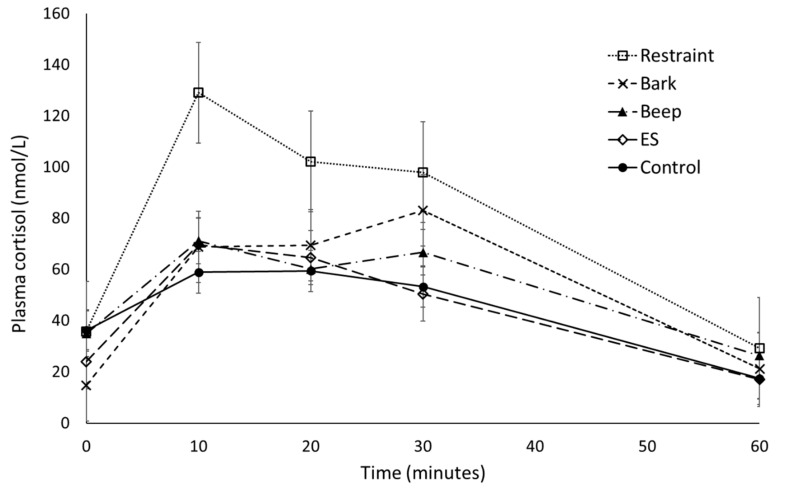
The effect of physical and auditory stimuli on mean (± s.e.m) plasma cortisol (nmol/L) in sheep. ES = electrical stimulus treatment.

There was no significant effect of treatment on the cortisol response area under the curve (F_4/71_ = 1.642, *p* = 0.173, Table 2), but there was a significant effect of cohort (F_3/71_ = 9.625, *p* ≤ 0.001). All animals had returned to baseline cortisol concentrations by 60 min from treatment time. There was a significant effect of cohort (F_4/78_ = 7.296, *p* = 0.009), a significant effect of time (F_1/314_ = 18.867, *p* ≤ 0.001), and a significant effect of baseline (F_1/314_ = 46.036, *p* ≤ 0.001) but no significant effect of treatment by time interaction (F_4/310_ = 0.758, *p* = 0.553) on the cortisol over time response over the 60 min following treatment. There was a significant effect of treatment on peak cortisol (F_4/315_ = 4.618, *p* = 0.001, Table 2) and a significant effect of cohort (F_1/78_ = 7.308, *p* = 0.008), with average peak occurring 10 min following treatment. There was a significant effect of cohort on overall mean plasma cortisol (average value from time 0 to 60) and change from baseline (F_3/72_ = 12.236, *p* ≤ 0.001 and F_3/72_ = 8.356, *p* ≤ 0.001, respectively), but no significant effect of treatment (F_4/72_ = 1.635, *p* = 0.175 and F_4/72_ = 2.411, *p* = 0.057, respectively). Average cortisol concentration for restraint, electrical stimulus, and control treatments peaked at 10 min following treatment, while beep and bark profiles demonstrated a small decrease at the 20 min sampling point with peak cortisol occurring 30 min following the treatment (Figure 1). 

#### 3.1.2. Vaginal Temperature

**Figure 2:**
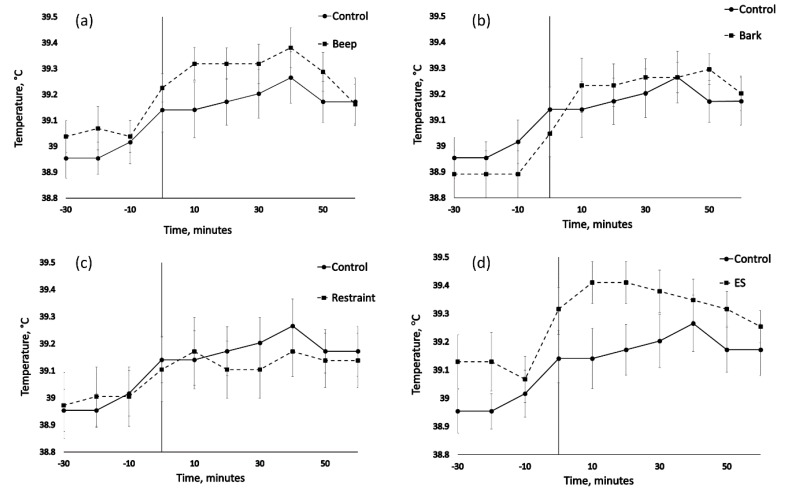
The effect of physical and auditory stimuli on mean (± s.e.m) vaginal temperature (°C) in sheep. Control comparison to treatments (**a**) beep, (**b**) bark, (**c**) restraint, and (**d**) ES = electrical stimulus treatment.

Mean, peak and change in vaginal temperature for each animal from time 0–60 (Table 2) showed no significant effect of treatment (F_4/71_ = 1.200, *p* = 0.333, F_4/71_ = 1.200, *p* = 0.305, F_4/71_ = 1.799, and *p* = 0.139, respectively) but did show a significant effect of cohort for mean and peak but not change (F_3/71_ = 5.300, *p* = 0.003, F_3/71_ = 5.6, *p* = 0.002, F_3/71_ = 1.777, and *p* = 0.159, respectively). Rank analysis of 5 min average data for time 0–60 showed no significant treatment differences in change from baseline (H(4) = 6.347, *p* = 0.175) for Kruskal-Wallis test and subsequent multi-comparison (α = 0.05). Time point analysis for time points 0 min, 10 min, 20 min, 30 min, 40 min, 50 min and 60 min from treatment showed no significant treatment differences (H(4) = 5.483, *p* = 0.241, H(4) = 5.638, *p* = 0.228, H(4) = 7.660, *p* = 0.105, H(4) = 6.075, *p* = 0.194, H(4) = 4.977, *p* = 0.290, H(4) = 4.511, *p* = 0.341, H(4) = 1.048, and *p* = 0.902, respectively for Kruskal-Wallis test and subsequent multi-comparison (α = 0.05)). Change from baseline analysis at time points 0 min, 10 min, 20 min, 30 min, 40 min, 50 min, and 60 min from treatment showed no significant treatment differences (H(4) = 2.316, *p* = 0.678, H(4) = 6.131, *p* = 0.190, H(4) = 6.667, *p* = 0.155, H(4) = 6.145, *p* = 0.189, H(4) = 3.068, *p* = 0.546, H(4) = 5.915, *p* = 0.206, H(4) = 4.479, and *p* = 0.345, respectively for Kruskal-Wallis test and subsequent multi-comparison (α = 0.05)). Temperature profiles showed little within individual variation with a mean range of 0.49 °C ± 0.03 s.e.m (Figure 2).

### 3.2. Behavioral Measures (Excluding Restraint Treatment)

#### 3.2.1. Vigilance Behaviors

There was a significant difference between the control treatment and the beep and bark treatments during both treatment (H(3) = 28.359, *p* ≤ 0.001) and post-treatment (H(3) = 25.512, *p* ≤ 0.001) observation periods, with beep and bark treatments spending a higher proportion of time being vigilant compared to the control treatments (Table 3). There was no significant difference between the electrical stimulus and control treatments for either observation period (*p* > 0.05) (Table 3). Jonckheere’s test showed a significant trend in the data for the treatment observation period (*J* = 515, *p* = 0.002) and the post-treatment observation period (*J* = 406.5, *p* ≤ 0.001).

#### 3.2.2. Ear Positions 

Significant differences were found in the treatment observation period in the duration of ear positions between the electrical stimulus and beep treatments for forward facing ears (*H*(3) = 9.342, *p* = 0.025) and backward facing ears (*H*(3) = 15.806, *p* = 0.001), and between the electrical stimulus and control groups for backward facing ears (*H*(3) = 15.806, *p* = 0.001). No significant differences were found between the treatments for ear positions planar and axial (*p* > 0.05). In the post-treatment observation period the beep treatment displayed a higher proportion of time with ears forward than the control (*H*(3) = 9.8633, *p* = 0.020) and the control treatment displayed a higher proportion of time with ears planar than the beep and bark treatments (*H*(3) = 19.282, *p* ≤ 0.001). There were no significant differences between treatments for ears facing backwards or axial in the post-treatment observation period (*p* > 0.05) (Table 3). Jonckheere’s test showed no significant trend in the data for the treatment observation period or the post-treatment observation period (*p* > 0.05).

The frequency of ear position change showed no significant differences between the treatments in the treatment observation period (F_3/57_ = 0.577, *p* = 0.6324), but during the post-treatment observation period, the beep treatment had a significantly lower number of ear position changes than the control treatment (F_3/57_ = 3.997, *p* = 0.012, Table 3).

#### 3.2.3. Point Behaviors

The number of steps taken were significantly different from the controls for all of the treatment animals (*p* ≤ 0.001), with bark and shock treatments showing a higher number of steps taken during the treatment observation period (z = 482.161, *p* ≤ 0.001 and z = 4.116, and *p* ≤ 0.001, respectively) and beep treatment showing a lower number of steps (z = −14.377, *p* ≤ 0.001). There was a significant difference between the beep treatment and the control treatment during the post-treatment observation, with the beep treatment taking a lower number of steps during this period (z = −2.156, *p* = 0.031) (Table 3).

Exploratory behaviors showed no significant effect of treatment during the treatment observation period (*p* = 0.238), but did show a significant overall treatment effect of during the post-treatment observation period (*p* = 0.002), with control and electrical stimulus treatment showing a higher number of exploratory behaviors than the other treatments, but multiple comparisons only tended toward significance (*p* (adjusted) = 0.053 (Table 4). 

There was a significant effect of treatment during both the treatment (*p* ≤ 0.001) and post-treatment (*p* ≤ 0.001) observation periods for avoidance behaviors. The electrical stimulus treatment showed a significantly higher number of avoidance behaviors than the other treatments during the treatment observation period (*p* ≤ 0.001). The bark and electrical stimulus treatments both showed significantly higher numbers of avoidance behaviors compared to control and beep treatments during the post-treatment (*p* = 0.009) observation period (Table 4). In particular the electrical stimulus treatment showed a number of novel behaviors during the treatment period which were not observed during the other treatments, such as hunching and stiff neck display, which were characterised as avoidance behaviors, as well as more extreme avoidance behaviors such as rearing and jumping, which were also not observed during the other treatments.

The elimination behaviors showed significant treatment effects (*p* = 0.012) in the treatment observation period. Control animals showed higher numbers of elimination behaviors and electrical stimulus animals showed higher numbers of shaking behaviors however these differences only tended towards significance when multiple comparison analysis was performed (*p* (adjusted) = 0.130 and *p* (adjusted) = 0.303, respectively). There were no significant treatment differences found during the post-treatment observation period (*p* > 0.05) (Table 4).

## 4. Discussion

As predicted, the responses to the audio stimuli suggest that the beep stimulus were assessed as benign, and did not illicit indicators of negative welfare except for increased vigilance. It was observed that the bark stimulus elicited increased avoidance behaviors and could therefore be considered an aversive audio stimulus, however, neither audio stimuli elicited a cortisol response that differed to the control treatment. A cortisol response is indicative of stress [15], while behavioral responses can indicate either stress or aversion, or both. Aversive responses in and of themselves may not indicate that the event was stressful to the animal. The physical (electrical stimulus and restraint) stimuli were expected to generate negative welfare states and this was observed in the sheep’s response. The high number of avoidance behaviors observed for the electrical stimulus suggests that a negative affective state was generated in response, and that this stimulus was more aversive than the dog bark stimulus. Restraint behaviors were not able to be observed to offer an equivalent comparison between the physical stimuli, however cortisol response to this treatment was consistent with previous research outlining stress responses to handling and restraint [10,34]. While aversive, the electrical stimulus did not elicit a significant cortisol response in comparison to the control treatment. However, the restraint treatment induced a higher cortisol response than the control and electric shock treatment, indicating that the electric shock was less stressful and potentially less aversive than one minute of restraint. The responses observed appear consistent with the hypothesis, in which stimuli may be ranked in order of increasing aversiveness and stressfulness from least/not aversive control, to the mild response elicited by the beep, a more aversive response to the bark stimulus, to the electrical stimulus eliciting a short lived, but strong aversive response, and finally the highly aversive and moderately stressful restraint procedure. 

In virtual fencing, a positive punishment stimulus is needed (in this case we use an electrical stimulus) for avoidance learning to occur. The positive punishment needs to be effective at training the desired behaviors (of staying within the boundary) by being aversive but not noxious, and the auditory cue should be benign in order to reduce stress and enable effective associative learning. The results of this study provide evidence of the relative aversiveness of the stimuli when first encountered during the initial learning process. When considered within the welfare assessment framework described in Lee, Colditz, and Campbell [6], during initial learning, the animals would be in a situation of low predictability and controllability (P/C) and negative affect. However, once animals learn to respond to the audio cue, the level of P/C would increase and affect would likely move to a more positive state. Situations of low P/C are commonly experienced by animals in their daily lives, and if short-term, the animal is able to cope. It becomes a welfare issue if the situation of low P/C is ongoing. This is when negative states such as helplessness and hopelessness can result, with serious implications for animal welfare [6]. 

Predictability of aversive stimuli is important in the context of animal welfare. An animal experiences poorer welfare when it is unable to predict when it may experience an aversive stimulus [8,35], and the uncontrollability of aversive events also showed this effect [36]. Conversely, the ability to control aversive stimuli reduced the indications of the effects of poor welfare in those animals [8], with impacts of both short term stress [37], and long term stress [38] on animal health and wellbeing. Therefore, it is important to view the results of this experiment in the context of short-term stress, and given that it is representative of the beginning of virtual fencing training. The animal’s perception is likely to change as predictability and controllability improves through the learning process. 

### 4.1. Physiological Responses

#### 4.1.1. Audio Stimuli

The lack of a significant cortisol or body temperature response to either of the audio treatments (beep and bark) indicates that neither of these two stimuli were aversive enough to elicit a physiological response, indicative of a significant stress event. It has been speculated that an aversive experience may not necessarily elicit a cortisol stress response unless the animal perceives it as aversive [39]. 

Duration of stimuli may have impacted on the intensity of the stress response exhibited: the bark duration was applied within a similar timeframe to the beep to offer a comparison. However, it is known that longer exposure to a barking dog, along with the visual presence of the dog, results in strong cortisol responses [13,14] but may not necessarily initiate stress induced hyperthermia [40], as was observed in the body temperature analysis for this study, in which no evidence for stress induced hyperthermia was found.

#### 4.1.2. Physical Stimuli

Plasma cortisol showed no significant difference between the control and electrical stimulus, however the restraint treatment animals showed a significantly higher cortisol concentration, showing that the restraint procedure was more aversive to the animal than the electrical stimulus. The duration of stress is likely to have impacted on the strength of the cortisol response observed. The electrical stimulus was applied for a shorter time period (less than 10 s to reflect existing protocol) than the restraint stimulus (one minute, reflective of normal husbandry practice). To reflect the virtual fencing training protocol, outlined in Marini, Meuleman, Belson, Rodenburg, Llewellyn, and Lee [5], and the electrical stimulus treatment was of very short duration, around ten seconds in total. While this was necessary in order to follow established protocols, the duration of the treatments is likely to have impacted on the strength of the cortisol response observed in this experiment. 

Intensity of stress is a consideration that may impact on cortisol responses. It is well known that even short-term isolation from peers is highly stressful to sheep [37], while other stressors are less aversive, such as the presence of a human when compared to the presence of a dog [41]. The observed results are consistent with previous work on restraint [10] and suggests that the electrical stimulus is less aversive than the commonly used restraint procedure in sheep. It is likely that the significant cohort effect observed in the body temperature measures is due to cohorts measured on different days and the ambient temperature variation across the days. 

### 4.2. Behavioral Responses

Behavioral responses were not able to be obtained for the restraint treatment due to operational difficulties. The short duration of behavioral responses observed for the other treatments may impact on the interpretation of these results, however, as the rate of a number of these behavioral responses changed between the treatment and post-treatment measurement periods, interpretations made are in consideration of those trends.

#### 4.2.1. Audio Stimuli

Sheep possess a high degree of auditory acuity [11], however, their perception of direction from which a sound originates is not as acute [42]. The perception of audio cues may rely on accompanying visual cues for contextual significance, and this visual information may be gained through vigilance behavior. Sheep exhibit vigilance behavior according to their perceived risk of predation [43,44] with a head lift and scanning of the environment [45] that may also, therefore, be behavioral indicators of affective state [46,47].

No sheep in the beep or bark treatments displayed exploratory behaviors in either the treatment and post-treatment observation periods, which is reflective of their high state of vigilance and is consistent with earlier work showing reduced exploratory behaviors in the presence of a dog [13,41]. Exploratory behaviors are generally exhibited as resource-seeking behaviors [13] and may also be considered as an indicator of a positive welfare state [48].

The bark treatment elicited a higher number of steps taken which may be an attempt at orienting behavior in the absence of visual stimuli [49], and a higher number of avoidance behaviors than the beep treatment during both treatment and post-treatment observation periods. This suggests that the sheep may have been more agitated by the bark (responding with attempts to flee) and perceived it as a more aversive sound, while the beep did not result in the same response. 

More elimination behaviors were shown during the control treatment than the beep and bark treatments, however these differences did not persist into the post-treatment period. While urination and defecation are associated with stress, it has also been observed that sheep fail to urinate in the presence of a dog and this may be due to the sheep attempting to avoid drawing the attention of a predator through olfactory cues [13]. 

Ear posture activity may be indicative of increased or decreased attention and may therefore be an inherent component of emotional reaction, with novel stimuli being initially interpreted as potentially negative until the animal obtains more information about its situation [50], and this may explain the reduction in ear position changes during the beep treatment when compared to the control treatment. However, use of ear postures in determining emotional state in sheep is still being researched. Previous research suggests that higher proportions of backward facing ears may be associated with positive events [50,51] in sheep, while other work suggests the opposite [20]. Reefmann et al. [52] found that the highest numbers of ear posture changes were observed in a negative situation rather than in a positive one, and this was assumed to indicate a negative emotional response in the sheep. In this experiment the assumed aversive treatment of the dog barking did elicit a higher proportion of axial ear postures, however this difference was not significant between treatments. 

Reefmann, Muehlemann, Wechsler and Gygax [52] found the proportion of asymmetric (axial) ear postures which were highest during a negative treatment, and this is consistent with current findings with the highest proportion of time spent displaying axial positioned ears during the bark treatment period. However, this had reduced to non-significance during the post-treatment observation period. More work is needed to provide sufficient evidence for the use of this measure in animal welfare assessments [51].

The behavioral responses by the animals to the beep and bark suggest that the beep is a benign audio stimulus, merely eliciting an attentive response. The bark stimuli appeared to elicit a stronger and longer lasting agitation and negative emotional response post-treatment. The physiological measures suggest that sheep did not experience significant stress.

#### 4.2.2. Physical Stimuli

Behavioral data were not available for the restraint treatment, and therefore only the responses to the electrical stimulus are discussed, however plasma cortisol results found are consistent with previous research on short term restraint in sheep, suggesting it is a moderately stressful procedure [34]. Analysis showed marked variation in the behavioral responses for the electrical stimulus treatment across individuals, with a wide range of avoidance behaviors displayed during both the treatment and post-treatment observation periods. This variable response to the electrical stimulus is consistent with responses observed in initial trials [5] with sheep experiencing the virtual fencing electrical stimulus for the first time. The range and number of avoidance behaviors exhibited reduced during the post-treatment observation period, suggesting that this effect may not be long lasting.

Exploratory behavior was not displayed by sheep in response to the electrical stimulus treatment during the treatment observation period. In the post-treatment observation period, the electrical stimulus treatment sheep exhibited a similar number of exploratory behaviors to the control treatment. This may indicate that the treatment did not have a long lasting impact, however the number of avoidance behaviors persisting suggests that a negative emotional state may still prevail in the post-treatment period. Alternatively, the high number of exploratory behaviors exhibited could indicate the desire to seek more information or reorient themselves. 

During the treatment observation period, higher proportions of backward facing ears were displayed by the electrical stimulus treatment animals compared to control animals. The electrical stimulus treatment elicited a higher number of ear posture changes, which reduced in the post-treatment observation period. This suggests that, while the stimulus may be aversive during application, its aversive effects were short-lived. 

Several notable novel behaviors were exhibited during the electrical stimulus treatment, including stiff neck, jumping, rearing, and falling behaviors that were not displayed at all during any of the other treatments. This suggests that this novel physical stimuli is eliciting reactions which are unique to this type of treatment, and may be analogous to escape attempts, but due to the treatment area being an enclosed space, there were no avenues of escape. These marked behaviors may indicate a negative emotional perception of the stimuli upon first interactions with it, however they appear to be short-lived responses as they did not persist beyond the treatment observation period into the post-treatment period. No behavioral data was available for the restraint treatment, but restraint and shearing are known to be highly aversive for sheep [10], and this was clearly shown in the cortisol response for this treatment. 

Importantly, it is noteworthy to reiterate that the electrical stimulus did not generate a significant increase in cortisol following treatment, suggesting that while the behavioral responses showed a strong negative response to the stimuli, this state was not of sufficient duration or intensity to trigger a change in plasma cortisol. Welfare outcomes for the treatments were varied, and must be viewed in the context in which they may be perceived by the animal. For example, the dog bark is not a novel stimulus and already has context for sheep that have been herded using dogs, and therefore is likely to have a negative association with it, as has been shown in previous work by Beausoleil [13]. Similarly, the restraint is not a novel stimulus, and associative learning may also be playing a role in influencing the perception of this stimulus for sheep, being associated with shearing and other stressful procedures that require restraint. Both the dog bark and restraint procedures may then be considered to demonstrate high predictability and low controllability, and may consequently impact on their affective state. These differences may also explain the variability in the responses, particularly to the electrical stimulus, which is an entirely novel stimulus with no context or previous associations, in addition to individual physiological differences affecting body temperature, cortisol and behavior [40,51]. 

This experiment was designed to assess the aversiveness of the stimuli used for virtual fencing at the beginning of the learning process when stimuli are novel, and where these stimuli may sit with respect to the predictability/controllability ratio. The high number of avoidance behaviors exhibited during the electrical stimulus treatment highlights the importance of evaluating the learning process via the welfare assessment framework described by Lee, Colditz, and Campbell [6], in order to reduce the number of electrical stimuli received. It also suggests that this stimulus is sufficiently aversive for the purpose of avoidance training. Successful learning will ensure that encounters with the aversive electrical stimuli will reduce over time, and thus avoiding it becoming a long-term stressor.

Planned future experiments involve the measurement of responses to stimuli in the context of the learned experience and training. This will examine how animals move within and across sectors of the welfare framework as the stimuli become familiar and both predictable and controllable and the subsequent effect of learning on affective states. These findings provide evidence to support the application of the welfare assessment framework as it may be applied to animals experiencing new technologies for the first time. 

## 5. Conclusions

It was predicted that the beep stimulus would be perceived as benign by the sheep when compared to the bark audio stimulus, and this has been confirmed through both physiological and behavioral responses to the stimuli. For the physical stimuli, it was predicted that the electrical stimulus would be less aversive than the restraint stimulus, but still elicit a strong negative affective state. The physiological responses supported this hypothesis, although there were several novel avoidance behaviors observed in response to the electrical stimulus. Within the context of the welfare framework outlined in Lee, Colditz, and Campbell [6], all treatments except the control are low in predictability and controllability and would therefore result in negative welfare if these conditions were to persist. However, with appropriate training and successful learning the predictability and controllability is likely to change and may affect perception of these stimuli. Following training, a welfare assessment should be undertaken to assess physiological and behavioral responses to these stimuli experienced by trained animals that are able to control and predict the stimuli. The results of this study suggest that these stimuli may be ranked within this framework from least aversive to most aversive as follows: control, beep, bark, electrical stimulus, restraint. This information can inform developers and users of virtual fencing systems and their application, and may also be of relevance to other emerging animal management technologies in which animal learning and welfare may be implicated. 

## Figures and Tables

**Table 1:** Behavioral ethogram.

Parameter of Behavior	Definition of Behavior (Characterisation)	Point or Continuous Sampling
Vigilance	Vigilant—head above shoulder (Vigilance)	Continuous
	Not vigilant—head parallel to or below shoulder height	
Ear posture	Ears in different directions (Axial)	Continuous
	Both ears facing backward (Backward)	
	Both ears neutral position (Planar)	
	Both ears facing forward (Forward)	
Elimination	Urination (Elimination)	Point
	Defecation (Elimination)	
Movement	One step of each leg or pair of legs (Step)	Point
Rapid movement	Run—rapid movement (Avoidance)	Point
	Step back—step backwards (Avoidance)	
Sniffing	Sniffing other sheep (Exploratory)	Point
	Sniffing ground (Exploratory)	
	Sniffing surroundings (Exploratory)	
Huddling	Huddling—grouping together so they are in close body contact (Avoidance)	Point
	Hiding—lowered head in corner or under other sheep (Avoidance)	
Avoidance responses	Jump—all four feet off the ground (Avoidance)	Point
	Rear—two feet off the ground (Avoidance)	
	Fall—quarters touching the ground (Avoidance)	
Shaking	Shaking head (Shaking)	Point
	Shaking body (Shaking)	
Avoidance novel behaviors	Stiff neck—observed as a stretching and rigidity of the neck posture (Avoidance)	Point
	Hunching—hunched back posture (Avoidance)	

**Table 2:** Mean, peak and change from baseline (± s.e.m) vaginal temperature and plasma cortisol measurements in the 60 min following treatment. Significant effects are as compared to control treatment. ES = electrical stimulus treatment.

Treatment	Vaginal Temperature (°C)			Plasma Cortisol (nmol/L)		
	Mean	Peak	Change from Baseline	Mean	Peak	Change from Baseline
	Mean ± s.e.m.	Mean ± s.e.m.	Mean ± s.e.m.	Mean ± s.e.m.	Mean ± s.e.m.	Mean ± s.e.m.
Control	39.2 ± 0.07 ^a^	39.4 ± 0.09 ^a^	0.5 ± 0.07 ^a^	53.5 ± 6.75 ^a^	93.9 ± 12.30 ^a^	77.0 ± 10.85 ^a^
Beep	39.2 ± 0.06 ^a^	39.4 ± 0.06 ^a^	0.5 ± 0.03 ^a^	49.7 ± 7.71 ^a^	84.4 ± 13.01 ^a^	71. 8 ± 12.14 ^a^
Bark	39.1 ± 0.07 ^a^	39.4 ± 0.09 ^a^	0.5 ± 0.05 ^a^	50.7 ± 7.86 ^a^	97.6 ± 15.16 ^a^	87.9 ± 13.90 ^a^
Restraint	39.1 ± 0.10 ^a^	39.3 ± 0.11 ^a^	0.4 ± 0.08 ^a^	84.5 ± 13.28 ^a^	155.4 ± 22.56 ^b^	130. 8 ± 17.97 ^b^
ES	39.3 ± 0.07 ^a^	39.5 ± 0.07 ^a^	0.4 ± 0.06 ^a^	47.9 ± 6.96 ^a^	80.2 ± 9.59 ^a^	66.9 ± 8.58 ^a^
*p*	ns	ns	ns	ns	0.08	ns

Data are means ± s.e.m., *n* = 16. Within a column, means not sharing a common letter differ (a, b), ns not significant.

**Table 3:** Effect of physical (ES) and auditory (beep and bark) stimuli on behaviors during observation period. ES = electrical stimulus treatment.

**Behavior**	**Treatment**				
	**Control**	**Beep**	**Bark**	**ES**	***p***
	**µ ± s.e.m.**	**µ ± s.e.m.**	**µ ± s.e.m.**	**µ ± s.e.m.**	
**Vigilance (%)**	62 ± 9.8 ^a^	100 ± 0.3 ^b^	99 ± 0.7 ^b^	82 ± 8.0 ^a^	***
**Ears axial (%)**	15 ± 5.0 ^a^	16 ± 4.6 ^a^	22 ± 3.4 ^a^	17 ± 3.7 ^a^	ns
**Ears planar (%)**	23 ± 8.9 ^a^	6 ± 2.6 ^a^	4 ± 1.5 ^a^	9 ± 2.5 ^a^	ns
**Ears forward (%)**	51 ± 11.2 ^ab^	70 ± 7.6 ^b^	58 ± 6.3 ^ab^	31 ± 8.0 ^a^	*
**Ears backward (%)**	12 ± 6.4 ^a^	9 ± 3. 8 ^a^	16 ± 4.3 ^ab^	43 ± 7.3 ^b^	***
**Ear posture changes (count)**	3 ± 0.5 ^a^	3 ± 0.5 ^a^	5 ± 0.5 ^a^	5 ± 0. 7 ^a^	ns
**Steps taken (count)**	1 ± 0.4 ^a^	1 ± 0.7 ^b^	5 ± 0.7 ^b^	5 ± 0.8 ^b^	***
**Behavior**	**Post-treatment**				
	**Control**	**Beep**	**Bark**	**ES**	***p***
	**µ ± s.e.m.**	**µ ± s.e.m.**	**µ ± s.e.m.**	**µ ± s.e.m.**	
**Vigilance (%)**	73 ± 7.8 ^ac^	98 ± 1.5 ^bc^	100 ± 0.0 ^b^	55 ± 10.3 ^a^	***
**Ears axial (%)**	14 ± 3.1 ^a^	9 ± 2.6 ^a^	13 ± 2.9 ^a^	15 ± 4.1 ^a^	ns
**Ears planar (%)**	13 ± 2.6 ^b^	2 ± 1.7 ^a^	1 ± 0.6 ^a^	6 ± 2.3 ^ab^	***
**Ears forward (%)**	57 ± 5.7 ^a^	82 ± 4.8 ^b^	75 ± 4.4 ^ab^	59 ± 8.6 ^ab^	*
**Ears backward (%)**	17 ± 4.5 ^a^	7 ± 3.1 ^a^	12 ± 2.7 ^a^	21 ± 7.1 ^a^	ns
**Ear posture changes (count)**	6 ± 0.7 ^a^	3 ± 0.6 ^b^	5 ± 0.7 ^a^	4 ± 0.8 ^a^	*
**Steps taken (count)**	4 ± 0.8 ^b^	2 ± 0.8 ^a^	2 ± 0.6 ^b^	4 ± 0.5 ^b^	*

Data are means ± s.e.m., *n* = 16. Within a row, means not sharing a common letter differ (a, b, c) (*, *p* < 0.05, *** *p* < 0.005, ns not significant).

**Table 4:** Counts of point behaviors displayed during treatment and post-treatment observation periods for Control, Beep, Bark, and Electrical Stimulus treatments (ES).

Treatment	Exploratory		Avoidance		Elimination		Shaking	
	Treatment	Post-treatment	Treatment	Post-treatment	Treatment	Post-treatment	Treatment	Post-treatment
Control	2 ^a^	6 ^a^	1 ^a^	1 ^b^	5 ^a^	1 ^a^	1 ^a^	2 ^a^
Beep	0 ^a^	0 ^a^	1 ^a^	1 ^b^	0 ^a^	1 ^a^	0 ^a^	0 ^a^
Bark	0 ^a^	0 ^a^	5 ^a^	9 ^a^	0 ^a^	1 ^a^	0 ^a^	0 ^a^
ES	0 ^a^	5 ^a^	14 ^b^	9 ^a^	1 ^a^	1 ^a^	4 ^a^	0 ^a^
*p*	n.s	**	***	***	*	n.s	*	n.s

*, *p* < 0.05, **, *p* < 0.01, *** *p* < 0.005, n.s. not significant. Within a row, means not sharing a common letter differ (a, b) Groups with a significant overall treatment difference but no significant difference between treatments only tended toward significance in multiple comparison analysis.

## Supplementary Materials

The following are available online at http://www.mdpi.com/2076-2615/9/1/30/s1, Table S1. Kearton all results, Table S2: Temperature time series, Table S3: Plasma cortisol Time series.
